# Arterial Stiffness and Hemodynamics in Young Women: The Effects of Oral Contraceptive Intake and Physical Habits

**DOI:** 10.3390/ijerph18073393

**Published:** 2021-03-25

**Authors:** Carina Enea, Pernelle Laffetas, Aurélien Pichon, Nathalie Delpech

**Affiliations:** Laboratoire MOVE (EA6314), Faculté des Sciences du Sport, Université de Poitiers, 8 Allée Jean Monnet—TSA 31113, 86073 Poitiers CEDEX 9, France; pernellelaffetas@gmail.com (P.L.); aurelien.pichon@univ-poitiers.fr (A.P.); nathalie.delpech@univ-poitiers.fr (N.D.)

**Keywords:** arterial stiffness, oral contraceptive pill, physical activity

## Abstract

Oral contraceptive (OC) intake seems to be associated with increased central hemodynamics and arterial stiffness. Conversely, physical activity (PA) is known to induce benefits on vascular structure and function, suggesting that the negative effects of the OC pill could be counterbalanced by regular PA. The aim of this cross-sectional study was to determine (1) whether OC intake in young women is associated with higher values of hemodynamic parameters and arterial stiffness and (2) whether these negative effects could be counterbalanced by regular physical activity. Forty-nine young healthy women (21.9 years ± 2.1) were recruited and divided into 4 groups, depending on their hormonal status (OC users: OC+ or non-OC users: OC−) and their physical habits (active/inactive). Assessments of central hemodynamics (central blood pressure, Aix75) and pulse wave velocity (PWV) were performed using applanation tonometry. cBP was higher in OC+ vs. OC−, while PWV was similar between these two groups. No interaction between physical activity and hormonal status was observed for any of these variables. Nevertheless, PWV was lower in young active women compared with age-matched inactive women, suggesting that the positive effect of regular physical exercise on the cardiovascular system is already visible in the first years of women’s adulthood, whatever the hormonal status.

## 1. Introduction

Oral contraceptive (OC) pills are the most widely used form of contraception in Europe and North America (17.8% of women) and are taken by over 150 million women worldwide [1]. In part because OC are effective and safe for contraception, and due to the fact that premenopausal women have lower cardiovascular risk than age-matched men, there has been relatively little specific study evaluating the effect of OC use on specific markers of cardiovascular health. However, although it is well-known that endogenous sex hormones (particularly 17ß-estradiol) provide cardiovascular protection in young women [2], the exogenous hormones contained in OC could be associated with rare but serious cardiovascular events, mainly due to thrombolic complications [3]. This harmful cardiovascular impact of OC results from the effect of exogenous estrogen (ethinyl-estradiol) on hemostatic factors (increasing coagulation factors, decreasing platelet aggregation and altering the lipid profile) which favor thrombosis [4]. Nevertheless, the prevalence of these thrombolic complications is highly dependent on other risk factors such as smoking, age and obesity [5]. On the same token, a recent meta-analysis including over 250,000 women found a significant association between duration of OC use and risk of hypertension [6]. Several pathophysiological mechanisms, such as oxidative stress [7], endothelial dysfunction, activation of the Renin-Angiotensin-Aldosterone System (RAAS) [8] and arterial stiffness [9] are thought to be implicated in this hypertensive effect.

Recently, specific attention has been paid to arterial stiffness, which is considered as an independent predictor of cardiovascular risk and mortality in the general population [10]. When the arteries stiffen, the transmission velocity of both the forward and the reflected waves increases, which causes the reflected wave to arrive earlier in the central aorta with greater amplitude and duration, leading to increased central systolic blood pressure (BP) and ventricular afterload, whereas diastolic BP decreases, resulting in reduced coronary perfusion [11]. Currently, the gold standard technique to determine arterial stiffness in humans is carotid-femoral Pulse Wave Velocity (PWV) measurement performed with tonometry applanation [12]. Exploration of the specific effects of sex hormones on arterial stiffness have started in the last few years. While it has been shown that prepubescent females have less compliant arteries (i.e., higher PWV) than their male counterparts, this trend is reversed after puberty [13], suggesting that 17ß-estradiol and testosterone affect the compliance of large arteries differently. In young premenopausal women, the hormonal status may also be of importance since some studies reveal an effect of the menstrual cycle and the OC use on central hemodynamics and/or markers of arterial stiffness [14]. However, discrepancies exist concerning the effects of OC use, which seem mainly linked to methodological considerations (i.e., different methods used to assess arterial stiffness, lack of control of the menstrual cycle phase and OC formulation, etc.) [15]. Indeed, Hickson et al. [9] have observed that OC users displayed significantly higher PWV than non-OC users, while another cross-sectional study reported higher values in peripheral and central BP, but not in PWV [16]. These results suggest that OC could exert structural changes on the arterial wall of the large arteries (i.e., increased elastin and decreased collagen and intima-media thickness), leading to increased arterial stiffness. Given the widespread use of OC and the lack of consensus concerning their effects on central hemodynamics and arterial stiffness, this issue is a major concern for women.

OC use is not the only factor that could affect cardiovascular health in premenopausal women. Several factors including lifestyle issues (physical inactivity, high salt intake, alcohol consumption, etc.) may strongly contribute to the pathogenesis of hypertension and cardiovascular diseases [17]. Conversely, regular physical activity is known to have benefits on vascular structure and function, resulting in a reduced risk of hypertension and cardiovascular diseases. Once again, this favorable effect is observed through several mechanisms, including oxidant/antioxidant balance, endothelial function [18] and RAAS [19]. Interestingly, physical exercise also has a positive effect on arterial stiffness, as recent studies performed on men and women have shown that physical activity is inversely related to various indices of arterial stiffness [20,21,22,23]. In normotensive premenopausal women, a 10-week resistance training is associated with a reduction of brachial-ankle PWV, which may be due to arterial structural changes [24]. The authors hypothesized that repeated mechanical distension of arteries induced by regular physical activity might stretch collagen fibers and modify their cross-linking, reducing arterial stiffness.

Based on the existing literature, we therefore hypothesized that (1) OC intake in young normotensive women is associated with higher values of hemodynamic parameters (brachial BP, central BP) and arterial stiffness (PWV) and (2) that these negative effects could be counterbalanced by regular physical activity (interaction between physical activity and hormonal status).

## 2. Materials and Methods

### 2.1. Subjects

A total of 49 young healthy women aged 18–28 years (mean: 21.9 ± 2.1 years) were enrolled in this study. Subjects were recruited via local university advertisements. Participants were divided into four groups, depending on their hormonal status (OC users (OC+) or non-OC users (OC−)) and their physical habits (active/inactive).

OC− had never taken hormonal contraceptives and reported regular menstrual cycles every 24–32 days (without variation > 3 days, from month to month). OC+ were women who had been taking a monophasic OC pill for at least 6 months before testing. While all oral contraceptives included consistent doses of ethynil estradiol (20–40 µg), they varied slightly as regards type of progestin (levonorgestrel, *n* = 21; norgestimate, *n* = 1, gestodene, *n* = 1 and chlormadinone, *n* = 2).

Women were classified as “active” or “inactive”, depending on their score on the Global Physical Activity Questionnaire (GPAQ) (Section 2.4).

None of the subjects presented heart disease, cardiovascular risk factors, renal disease, respiratory disease or inflammatory disease. As concerns inclusion criteria, subjects had to be nonsmokers, normotensive, with a body mass index (BMI) falling in the normal weight category (18.0–24.9 kg/m^2^), asymptomatic, and non-users of any medications that could interfere with the experimentation.

The sample size was calculated using G*Power software (version 3.1.9.7, Düsseldorf, Germany) with an effect size of 0.40, power of 0.80 and confidence level of 0.05. A total of 52 participants were required. The study was approved by a national ethics committee for non-interventional research (CERSTAPS). Written informed consent was obtained for all participants.

### 2.2. Study Design

**Figure 1:**
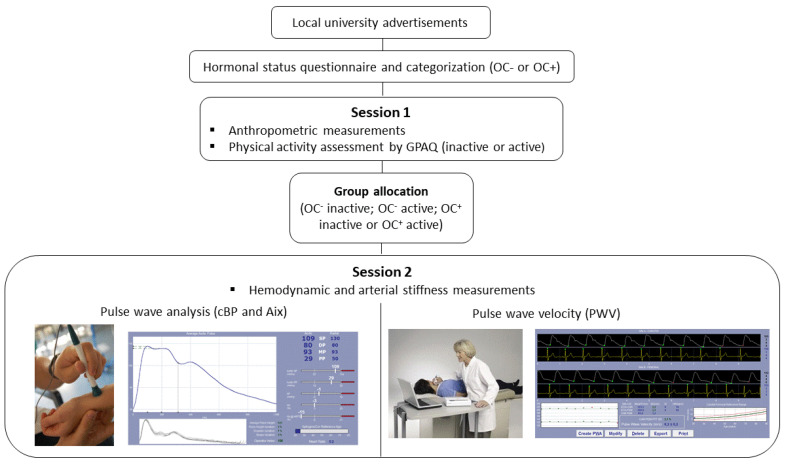
Enrollment of the study participants and study design.

Participants completed all the tests on two separate sessions. Session 1 included anthropometric measurements and physical activity assessment by the Global Physical Activity Questionnaire. Participants were then allocated in one of the following groups, depending on their hormonal status and their physical activity level (OC− inactive, OC− active, OC+ inactive or OC+ active). Session 2 included hemodynamic and arterial stiffness measurements and was performed during the early follicular phase of the menstrual cycle (day 4 ± 2) for OC− and during the active phase of OC regimen for OC+. Participants abstained from all caffeine-containing beverages and food, as well as ethanol intake, for at least 12 h, and from strenuous exercise for 24 h prior to this visit. The study design is presented in Figure 1.

### 2.3. Anthropometric Measurements

Height was measured via a Stadiometer (Tanita Leicester) in a standing position with shoes removed, shoulders relaxed and facing forward. Weight and total body fat (FM%) were measured via a Tanita TBF-310GS Total Body Composition Analyzer. These measurements were performed in the standing position, after bladder emptying.

### 2.4. Physical Activity Assessment

The duration and frequency of physical activity participation over a typical week were recorded using the interviewer-administered version of the Global Physical Activity Questionnaire (GPAQ) [25]. The French version of the GPAQ was approved as a means of measuring physical activity by Riviere et al. [26]. This questionnaire contains 16 items designed to assess the frequency and duration of PA during a typical week in 3 domains: work, transportation and leisure time. It distinguishes PA duration by min/day and min/week for each PA domain, which allows for calculation of energy expenditure in terms of metabolic equivalent of task (MET). One MET corresponds to resting energy expenditure. An estimate of total moderate-to-vigorous-intensity physical activity (MVPA, MET-min/week) was calculated by combining the score of both moderate and vigorous-intensity activities (according to GPAQ analysis guide [27]).

Participants were then classified as “active” or “inactive”, depending on their total MVPA per week (MET-min/week). Defined as “active” were those who met the following global recommendations of World Health Organization: 30 min of moderate-intensity activity or walking per day, during at least 5 days in a typical week; or 20 min of vigorous-intensity activity per day during at least 3 days in a typical week; or 5 days of any combination of walking and moderate- or vigorous-intensity activities attaining a minimum of at least 600 MET-min/week. Those who did not meet these criteria were classified as “inactive”.

### 2.5. Hemodynamic and Arterial Stiffness Measurements

All of these vascular function measurements were conducted according to recent recommendations by the American Heart Association [28].

#### 2.5.1. Brachial Blood Pressure

After 10 min of rest in a supine position, brachial blood pressure (bBP) measurements were performed in triplicate in the dominant arm, using an automated noninvasive blood pressure cuff (Mobil-O-Graph, I.E.M. GmbH, Stolberg, Germany). An average of three BP measurements was considered as resting BP value and was then used for the Sphygmocor calibration, which is the device employed in Pulse Wave Analysis (central BP and AIx) and Pulse Wave Velocity measurements.

#### 2.5.2. Pulse Wave Analysis (Central Blood Pressure and AIx Measurements)

Pulse Wave Analysis was performed by radial tonometry, with a high sensitivity Millar tonometer included with the Sphygmocor CVMS system (AtCor Medical, Sydney, Australia). As described below, radial tonometry waveforms were calibrated to brachial cuff systolic and diastolic pressures assessed immediately before testing. Radial tonometry waveforms were then used to estimate the following variables: central blood pressure (cSBP, cDBP); augmented pressure (AP; the difference between the first and second systolic shoulders of central systolic blood pressure, i.e., amplitude of the reflected wave); aortic augmentation index (AIx) and aortic augmentation index adjusted for a heart rate of 75 beats/min (AIx75). All of these hemodynamic variables were calculated with the Sphygmocor’s generalized transfer function.

#### 2.5.3. Pulse Wave Velocity (PWV)

Carotido-femoral PWV was measured from the common carotid pulse to the femoral pulse using the same equipment as PWA (applanation tonometry), in combination with three-lead electrocardiography (SphygmoCor, AtCor Medical, Sydney, Australia). PWV was automatically calculated from measurements of pulse transit time and distance between the two recording sites, carotid and femoral (PWV = distance (m)/transit time (s). Corrected PWV was then calculated by multiplying by 0.8 [29]. To ensure reliable measurement, the standard deviation of measurements had to be less than 6%.

### 2.6. Statistical Analysis

Data were analyzed using SPSS software (SPSS Statistics, IMB, Armonk, NY, USA). Normal Gaussian distribution of the data was verified by the Shapiro–Wilks test and homoscedasticity by a modified Levene Test. One-way ANOVA was used to compare anthropometric and physical activity (MVPA) data between groups. When applicable, the Bonferroni post-hoc test was performed to identify significant differences between groups.

For heart rate (HR) data, the respective influence of hormonal status and physical activity (as independent variables) were assessed by two-way ANOVA. As it is well-known that Fat Mass (%) influences hemodynamic and arterial stiffness parameters, these data (bBP, cBP, AIx75, PWV) were analyzed by two-way ANCOVA with two main effects (hormonal status x physical activity) with Fat Mass (%) as covariate to access the potential interaction effect of physical activity by hormonal status. When applicable, a post-hoc multiple comparison Newman-Keuls test was performed to identify significant differences between groups. The significance level for all analyses was set at *p* < 0.05.

The magnitude of the difference was then assessed by effect size (ES), which was calculated by dividing the difference of two means by the pooled SD; effect sizes were the classified as small (d = 0.2), medium (d = 0.5), large (d ≥ 0.8), or very large (d ≥ 1.2) [30].

## 3. Results

### 3.1. Participant Characteristics

One-way ANOVA analysis highlighted the differences between participant groups (Table 1). There was no difference between groups in age, weight or height. However, significantly higher BMI was observed in the OC+ inactive group compared with the OC− inactive (*p* < 0.001), OC− active (*p* < 0.05) and OC+ active (*p* < 0.05) groups. Fat mass% was higher only in OC+ inactive compared to OC+ active (*p* < 0.02). The two active groups (OC+ and OC−) presented significantly higher MVPA time per week than the two inactive groups (higher *p* value < 0.005) and no significant differences were observed between OC+ active and OC− active (*p* = 0.66).

### 3.2. Hemodynamic Parameters

**Figure 2:**
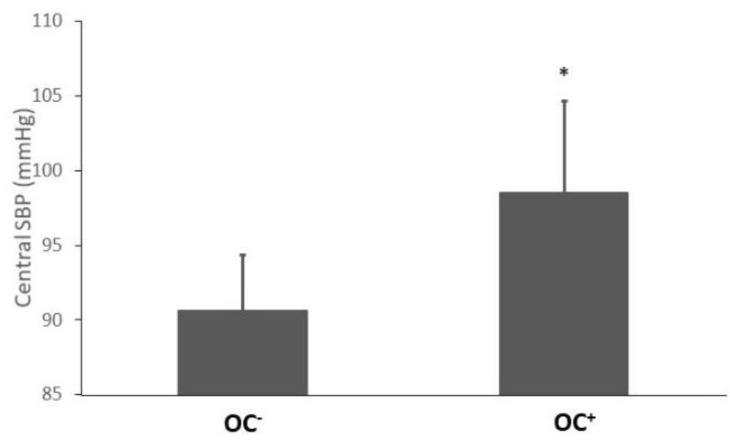
Central SBP in OC− and OC+ groups. All values are presented as mean ± standard deviation. Central SBP, central systolic blood pressure; * indicates significant difference between OC− and OC+ groups (OC main effect *p* < 0.000001).

**Figure 3:**
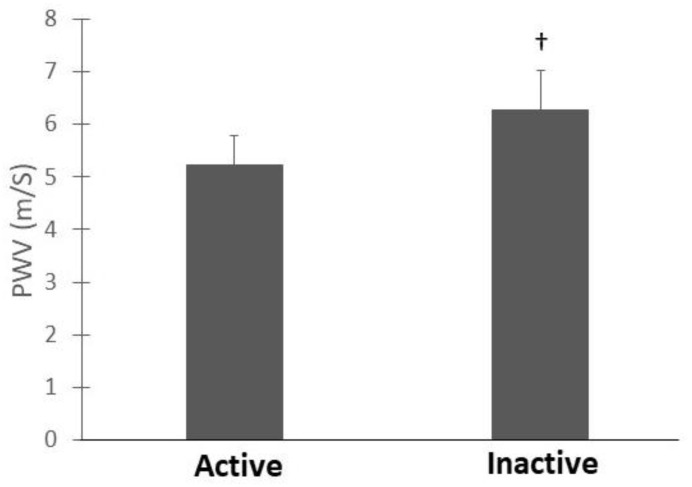
Pulse wave velocity in active and inactive groups. All values are presented as mean ± standard deviation. PWV, pulse wave velocity; † indicates significant difference between active- and inactive groups (physical activity main effect *p* < 0.0001).

Two-way ANCOVA (hormonal status × physical activity) with Fat Mass% as covariate analysis provided the results for hemodynamic parameters recorded in Table 2. No interaction between hormonal status and physical activity was observed, regardless of hemodynamic parameters. However, main statistical effects were observed for hormonal status and physical activity. These effects are detailed in Figure 2 and Figure 3.

Brachial and central SBP were higher in OC+ compared with OC−, independently of their physical habits ((bSBP OC+: 116.4 ± 6.1, OC−: 108.1 ± 3.8 mmHg, *p* < 0.0001, (d = 1.69); cSBP OC+: 98.6 ± 6.1, OC−: 91.6 ± 3.1 mmHg, *p* < 0.001, (d = 1.51), Figure 2)). On the contrary, AIx75 was higher in OC− compared with OC+ (−4 ± 12 and −11 ± 12, respectively, d = 1.25). No effect of hormonal status was found for PWV.

Heart rate at rest was significantly lower in active compared with inactive groups (active: 55 ± 8 bpm, inactive: 66 ± 9 bpm; *p* < 0.01; d = 1.26). Similarly, lower PWV values were found in active compared with inactive groups ((5.3 ± 0.4 vs. 6.4 ± 0.7 ms^−1^, respectively, *p* < 0.00001; (d = 1.82), Figure 3).

## 4. Discussion

The aim of the current study was to assess the effect of hormonal status (OC− vs. OC+) on central hemodynamics and arterial stiffness in young women, depending on their physical activity level (inactive vs. active). Based on the existing literature, we hypothesized that (1) OC intake in young women (OC+) is associated with higher values of hemodynamic parameters (brachial BP, central BP) and arterial stiffness (PWV) than in eumenorrheic women (OC−) and that (2) these negative effects could be counterbalanced by regular physical activity (interaction between physical activity and hormonal status). Our results partially support our first hypothesis, as we found that OC+ have higher brachial and central blood pressures than OC−. On the other hand, PWV, a strong marker of arterial stiffness, was not affected by OC use. This result is of importance, as it suggests that higher values of blood pressures in OC+ are not related to an enhancement of aortic arterial stiffness (PWV). As regards our second hypothesis, the absence of interaction between physical activity and hormonal status (OC− vs. OC+) and brachial and central blood pressure indicates that physical activity is not able to counterbalance the effect of OC intake. Nevertheless, the lower PWV observed in young active women compared with age-matched inactive women (independently of hormonal status), suggests for the first time the positive effect of regular physical exercise on arterial stiffness in this specific population. These results indicate that exogenous hormones and physical activity influence cardiovascular health markers in different ways, without interaction between these two independent variables.

### 4.1. Effect of Oral Contraceptives

As expected, we found significantly higher values of brachial SBP and DBP in OC+ compared with OC− (differences of 8.3 and 5.5 mmHg, respectively). These results are in line with previous studies that showed increased peripheral blood pressure in normotensive OC users [31], leading to an increased risk of hypertension in this population [32,33]. However, and even though brachial BP measurement is widely used in clinical practice, recent findings suggest that central BP is more pronouncedly related than brachial BP to future cardiovascular events and to the pathophysiology of end-organ damage [34]. Assessment of central hemodynamics seems particularly relevant in healthy young persons, as major disparities can be observed between central and brachial BP in this part of the population. These differences in young subjects are largely due to a phenomenon of systolic pressure amplification throughout the arterial tree [35]. For example, it has been shown that more than 30% of males and 10% of females with normal brachial BP had aortic pressure comparable to that of individuals with stage 1 hypertension [36]. Our study shows higher central hemodynamics (cSBP, cDBP, cPP) in OC+ than in OC−. Despite conflicting results existing concerning the effects of OC on central hemodynamics [9,15,16,37], our findings are in line with those of the CYCLIC study, in which OC users have significantly higher values in peripheral and central blood pressure than eumenorrheic age-matched control [16]. While the mechanisms leading to this blood pressure raise are still incompletely understood, evidence suggests that both of the exogenous hormones found in combined OC (ethinyl-estradiol and progestin) are implicated in this phenomenon. Interestingly, the risk of hypertension associated with OC increased with age, duration of use, body mass and progestin potency [38]. Exogenous hormones are known to influence several physiological mechanisms implicated in blood pressure regulation, including the renin-angiotensin-aldosterone system (RAAS), endothelial function, the sympathetic nervous system, oxidative stress [39], and arterial stiffness [9].

Indeed, in the present study we hypothesized that the hypertensive effect of OC could be partly mediated by increased arterial stiffness, as assessed by AIx75 and carotid-femoral PWV. We found that AIx75 was slightly better in OC+ compared to OC− (−11 ± 12 vs. −4 ± 12, respectively), while PWV was not affected by hormonal status. Once again, discrepancies exist concerning the effects of OC on different markers of arterial stiffness [9,15,16,37]. However, our results are in line with those of Yu and al., for whom OC use was associated with significantly higher values in aortic and peripheral blood pressure, but not in PWV [16]. Similarly, Priest and colleagues did not observe differences in PWV between OC users (2nd, 3rd, and 4th generation OC pill) and naturally cycling controls [15]. Taken together, these results suggest that higher values of blood pressures in OC+ are not related to an enhancement of aortic arterial stiffness (PWV). The fact that no significant correlation was observed between PWV and cSBP values in our OC+ group (r^2^ = 0.15) supports this hypothesis (data not shown).

In contrast, a statistically significant difference in PWV was found between OC users and non-users in a cross-sectional study involving 885 women (ENIGMA study) [9], but the small difference observed (0.1 m/s) is unlikely to be clinically significant. A number of factors, including study design, could account for these divergent findings. In the ENIGMA study, the types of progestins and the OC dosages were not specified and may partially explain the discrepant results. Moreover, eumenorrheic women were evaluated in different phases of their menstrual cycle, and it remains open to question whether the menstrual cycle can indeed affect arterial stiffness [15,16,40,41]. Furthermore, in our study we paid particular attention to standardizing the conditions and timing for evaluation in all subjects, and also endeavored to control potential interfering factors.

At first glance, the fact that OC can affect AIx75 without modifying PWV can appear surprising. However, although both of them are markers of arterial stiffness, AIx75 is used as a direct marker for wave reflection and an indirect marker for arterial stiffness, whereas PWV is considered a direct and ‘‘gold standard” marker of arterial stiffness [42]. To date, findings from the literature do not satisfactorily explain our results concerning the effects of OC on AIx75, as we expected higher values (or at least the same values) in OC users than in non-OC users. AIx75 is an integrative marker of cardiac and vascular properties, including forward-traveling pulse wave generated from LV ejection, large-artery stiffness and magnitude of the backward-traveling pulse wave generated from the reflecting properties of microcirculation [43,44]. Therefore, it would be interesting to quantify the effect of OC use on cardiovascular parameters that could influence AIx (e.g., stroke volume, ejection fraction, left ventricular contractility and peripheral resistance), in a normotensive population of young women.

As mentioned earlier, the exact mechanisms that lead to an increase in central SBP associated with OC use are not completely understood. However, it has been shown that exogenous estrogen could affect RAAS activity by increasing the circulating components of this system (angiotensinogen, ANG II, and aldosterone) [45]. The interactions between estrogen and progestins are complex and the effects of progestins on RAAS vary highly, depending on type of synthetic compound [46]. In fact, progestins derived from testosterone, progesterone or spironolactone may have different physiological effects on the cardiovascular system, according to their structure or metabolites [46]. For example, it was recently shown that drospirenone, a progestin derived from spironolactone, which has antimineralocorticoid diuretic effects, blunted the BP-increasing effect of OC use [47]. In our study, 23 of the 25 women of the OC+ group used an oral contraceptive containing progestins derived from testosterone (levonorgestrel, gestodene, norgestimate), which may explain the high values and the small standard deviation of the central SPB compared to other studies [9,16]. Prolonged used of low-dose OC may also modify endothelial function (assessed by flow-mediated dilation—FMD) [48,49,50], leading to higher blood pressure values in OC users compared with matched-age controls. Once again, the type of progestin is important since it has been shown that OC containing levonorgestrel is associated with more pronounced changes in FMD than an OC containing chlormadinone, a progestin derived from progesterone [50].

To conclude this part of the discussion, our results suggest that the higher central blood pressure observed in OC users is not related to an enhancement of aortic arterial stiffness (PWV) in this population. Further studies are needed to better understand the long-term clinical implications of this higher value and to determine the physiological mechanisms involved.

### 4.2. Effect of Physical Activity

In this study, we hypothesized that the negative effect of OC use in peripheral and central hemodynamics (supposedly mediated by increased arterial stiffness), could be counterbalanced by regular physical activity. We therefore expected to find an interaction between physical activity level (inactive vs. active) and hormonal status (OC− vs. OC+) for these parameters. Our study nonetheless failed to find any interaction between these two independent variables (hormonal status × physical activity), suggesting that regular exercise does not protect women from the slight BP raise observed with OC use. This lack of interaction is not surprising insofar as our results show similar PWV values between OC+ and OC−. However, this result raises the question of the mechanisms involved in the increase in blood pressure induced by contraceptives and on which physical activity does not seem to have an effect.

Interestingly, a significant difference was found between physically active and less active women (5.4 ± 0.6 vs. 6.3 ± 0.8 ms^−1^ for PWV, respectively), suggesting that the positive effect of regular physical exercise on large artery stiffening is already effective in the first years of adulthood. To our knowledge, only two studies performed on middle-age subjects (male and female) have shown that physical activity was inversely related to markers of arterial stiffness [20,23], whereas sedentary time was positively associated with the latter [23]. Although our results are in accordance with these previous data, the amplitude of the difference and the small standard deviation observed in our study are somewhat surprising. They could potentially be explained by the high level of MVPA in our active group, as the experimental subjects are sport science students. As was observed for PVW, a significant difference in resting heart rate was found between our inactive and active groups (66 ± 9 vs. 57 ± 8). This is in accordance with the literature, underlining a significant positive link between heart rate and arterial stiffness of large arteries [51]. Even though the mechanism by which exercise training ameliorates arterial stiffness is not completely understood, improved endothelial function has been postulated. Recently, Beck et al. [52] showed that short-term (8 weeks) exercise training can improve endothelial function and vasoactive balance in young prehypertensive subjects. Physical exercise is also known to reduce hypertension by decreasing elevated sympathetic nervous system (SNS) activity [53]. However, in normotensive subjects, the relationship between SNS activity and arterial stiffness is complex and appears to be sex- and age-dependent [54,55,56]. Although muscle sympathetic nerve activity is positively associated with central SBP and Aix in men [57] and older postmenopausal women [55], no relationship or even a negative relationship between these variables has been observed in young premenopausal women [57]. These results are supported by recent findings reporting that ganglionic blockade significantly reduces PWV in postmenopausal women but not in young women [54]. This suggests that the physiological mechanisms implicated in the positive-effect of exercise in women can vary considerably with age. Therefore, intervention studies should be carried out on young women, the objective being to better understand the mechanisms through which physical activity enhances arterial stiffness.

#### Strengths and Limitations

The present study had several strengths. To our knowledge, it is the first study to assess the combined effects of physical activity level and oral contraceptive intake on central hemodynamics and arterial stiffness in young women. Given the large number of women using OC worldwide, it is clinically relevant to assess whether the negative effects of OC on these markers could be counterbalanced by regular physical activity. The strengths of this study include the intra-group homogeneity due to the strict inclusion criteria. It probably explains the amplitude of the difference and the small standard deviation in PWV and cBP observed. The use of two-way ANCOVA in data analysis allows us to determine whether there was or not an interaction effect between hormonal status and physical activity on the variables tested, after taking into account the % of fat mass. Use of the GPAQ to stratify the physical activity level of our participants seems relevant insofar as this questionnaire covers several important components of physical activity, such as intensity, duration, and frequency. It also assesses three domains in which physical activity is performed (occupational physical activity, transport-related physical activity, and physical activity during discretionary or leisure time).

However, our study also has some limitations. As a result of its cross-sectional design, this study shows only differences between groups and cannot be used to propose causal relationships. Moreover, the small sample size of subgroups (11 ≤ *n* ≤ 13) necessitates caution in interpreting the results of the two-way ANCOVA. Although they provide interesting outcomes, they should be confirmed in a larger sample, with interventional and longitudinal study designs. Moreover, this study does not allow to determine the biological or physiological mechanisms explaining our results, especially the effect of regular physical activity on PWV. In our study, we did not perform a time-dependent analysis to assess the impact of duration of OC use and physical activity level on arterial stiffness and hemodynamic properties. However, one requirement for inclusion in the study was continuous low-dose OC use for a minimum of 6 months. Additionally, the self-reporting of physical activity level and menstrual cycle phase/OC use may lead to misclassification. The high MVPA level of our active group may not represent the active women who respect the current the American College of Sports Medicine and the American Heart Association guidelines (moderate-intensity physical activity for at least 30 min, 5 days a week or vigorous-intensity activity for at least 20 min at least 3 times a week to promote and maintain health [58].

## 5. Conclusions

Our study confirms that women using OC have heightened central BP when compared to non-users, although it is not known whether this difference is clinically meaningful and if it induces an increase of the long-term CVD risk in this population. Contrary to our initial hypothesis, our results do not suggest that a high level of physical activity is able to counterbalance the effect of OC intake. Nevertheless, this study shows for the first time that PWV in young active women (OC+ and OC−) is lower than in age-matched inactive controls, suggesting that the positive effect of regular physical activity on large arterial stiffness is already visible in the first years of women’s adulthood, whatever the hormonal status. These results are coherent with the growing body of evidences showing that physical activity is an effective strategy for primary prevention of cardiovascular diseases in women. Additional researches specific to this population are needed to better understand the factors that contribute to the modification of CVD risk during the women lifespan.

## Figures and Tables

**Table 1:** Participant characteristics.

Participant Characteristics	OC− Inactive (*n* = 11)	OC− Active (*n* = 13)	OC+ Inactive (*n* = 13)	OC+ Active (*n* = 12)	*p*	Differences
Age (years)	22.0 ± 2.9	20.5 ± 1.6	22.3 ± 2.4	21.5 ± 1.5	0.19	-
Weight (kg)	57.4 ± 6.2	59.4 ± 5.7	60.8 ± 6.6	58.2 ± 4.6	0.51	-
Height (m)	1.67 ± 0.1	1.67 ± 0.1	1.62 ± 0.1	1.66 ± 0.1	0.06	-
BMI (kg/m^2^)	20.5 ± 1.6	21.2 ± 1.4	23.1 ± 1.9	21.1 ± 1.7	*p* < 0.05	OC+I > OC−A, OC−I, OC+A
Fat mass (%)	23.6 ± 3.5	23.4 ± 4.4	26.2 ± 4.4	20.9 ± 5.0	*p* < 0.05	OC+I > OC+A
Total MVPA (MET-min/week)	281 ± 106	1816.2 ± 1175	293 ± 179	1340 ± 789	*p* < 0.05	OC−A > OC−I, OC+I OC+A *>* OC−I, OC+I
Cycle length, days	28.2 ± 2	29.3 ± 2				-

All values are presented as mean ± standard deviation. BMI, body mass index; MVPA, moderate-to-vigorous-intensity physical activity; OC+I, OC+ inactive; OC+A, OC+ active; OC−I, OC− inactive; OC−A, OC− active. OCs included ethynil estradiol (20–40 µg) and progestin (levonorgestrel, *n* = 21; norgestimate, *n* = 1, gestodene, *n* = 1 and chlormadinone, *n* = 2).

**Table 2:** Hemodynamic parameters.

	OC−		OC+		Main Statistical Effects	
Hemodynamic Parameters	Inactive (*n* = 11)	Active (*n* = 13)	Inactive (*n* = 13)	Active (*n* = 12)	Hormonal Status	Physical Activity
bSBP (mmHg)	107.5 ± 5.0	106.5± 3.3	115.1 ± 5.0	117.8 ± 7.0	*p* < 0.000001	0.64
bDBP(mmHg)	66.1 ± 3.8	63.2 ± 3.1	70.4 ± 4.5	71.1 ± 6.9	*p* < 0.00005	0.66
bMBP(mmHg)	85.3 ± 6.1	81.5 ± 6.1	92.7 ± 4.1	94.5 ± 6.6	*p* < 0.000001	0.54
bPP(mmHg)	41.5 ± 5.4	43.3 ± 2.7	44.7 ± 4.8	46.8 ± 4.5	*p* < 0.02	0.30
cSBP(mmHg)	91.9 ± 3.6	89.6± 3.5	97.7 ± 6.3	99.5 ± 6.1	*p* < 0.000001	0.90
cDBP(mmHg)	67.2 ± 3.9	64.3 ± 3.2	70.8 ± 4.8	71.3 ± 6.7	*p* < 0.001	0.62
cMBP(mmHg)	79.4 ± 3.5	76.5 ± 3.6	84.2 ± 5.2	85.4 ± 6.2	*p* < 0.0001	0.65
cPP(mmHg)	24.7 ± 3.3	25.3 ± 1.5	26.9 ± 4.1	28.2 ± 3.6	*p* < 0.001	0.58
HR (bpm)	68.5 ± 8.4	57.1 ± 8.9	64.5 ± 9.0	57.8 ± 8.3	0.50	*p* < 0.00001
AIx75	1.7 ± 7.8	−9.5 ± 12.0	−13.2 ± 13.0	−8.5 ± 9.8	*p* < 0.05	0.17
PWV (m/s)	6.4 ± 0.9	5.2 ± 0.6	6.2 ± 0.7	5.6 ± 0.5	0.49	*p* < 0.0001

All values are presented as mean ± standard deviation. bSBP, brachial systolic blood pressure; bDBP, brachial diastolic blood pressure; bMBP, brachial mean blood pressure; bPP, brachial pulse pressure; cSBP, central systolic blood pressure; cDBP, central diastolic blood pressure; cMBP, central mean blood pressure; cPP, central pulse pressure; HR, heart rate; AIx75, augmentation index adjusted at a heart rate of 75 bpm; PWV, pulse wave velocity.

## Data Availability

The datasets used and/or analyzed during the current study are available from the corresponding author on reasonable request.

## Abbreviations

AIx75	Augmentation Index adjusted at a heart rate of 75 bpm
AP	Augmented Pressure
BP	Blood Pressure
MAP	Mean Arterial Pressure
MET	Metabolic Equivalent Task
MVPA	Moderate-to-Vigorous-Intensity Physical Activity
OC	Oral Contraceptive
PP	Pulse Pressure
PWA	Pulse Wave Analysis
PWV	Pulse Wave Velocity
